# 12-year trends in cardiovascular risk factors (2002-2005 through 2011-2014) in patients with cardiovascular diseases: Tehran lipid and glucose study

**DOI:** 10.1371/journal.pone.0195543

**Published:** 2018-05-16

**Authors:** SeyedHossein Rabani, Mahsa Sardarinia, Samaneh Akbarpour, Fereidoun Azizi, Davood Khalili, Farzad Hadaegh

**Affiliations:** 1 Prevention of Metabolic Disorders Research Center, Research Institute for Endocrine Sciences, Shahid Beheshti University of Medical Sciences, Tehran, Iran; 2 Department of Epidemiology and Biostatistics, School of Public Health, Tehran University of Medical Sciences, Tehran, Iran; 3 Endocrine Research Center, Research Institute for Endocrine Sciences, Shahid Beheshti University of Medical Sciences, Tehran, Iran; University of Tampere, FINLAND

## Abstract

**Background:**

To examine the trend of cardiovascular diseases (CVD) risk factors among a Middle Eastern population with prevalent CVD during a median follow up of 12 years.

**Methods:**

Patients with prevalent CVD (n = 282, men = 167), with a mean age of 60.76 years were evaluated in four study phases of the TLGS (Tehran lipid glucose study), 2002–2005, 2005–2008, 2008–2011, and 2011–2014. Trends of CVD risk factors were estimated using generalized estimation equation (GEE) models, by adjusting for gender, age and propensity scores.

**Result:**

The adjusted prevalence of general and central adiposity, diabetes and physical inactivity at baseline was 25.18, 60.14, 25.03 and 43.74%, respectively and had increasing trends during the study period, reaching 41.32, 66.74, 43.20 and 50.32%, respectively, at the last visit. Although systolic, but not diastolic blood pressure, decreased from 134.88 to 129.86 mmHg, the prevalence of hypertension did not decrease (64.21% vs 68%, p value = 0.326). The prevalence of low high density lipoprotein cholesterol (HDL-C), hypertriglyceridemia and high non-HDL-C at baseline was 74.54, 59.89 and 96.53%, respectively, and showed improved trends reaching 44.87, 47.12 and 96.06% respectively; however, the favorable trend was not observed for high low density cholesterol. Significant increasing trends were observed in the consumption of anti-hypertensive, lipid and glucose lowering medications, but not for aspirin. The prevalence of current smoking (11.05 vs 16.83%, p value = 0.042) and chronic kidney disease (44.16 vs 51.65%, p value = 0.054) increased during follow up.

**Conclusion:**

Except for lipid profile status, dangerous trends for other CVD risk factors were demonstrated among CVD patients, which can be a harbinger for high rates of CVD mortality; these findings highlight the need for urgent implementation of multicomponent interventions to control CVD risk factors among these patients.

## Introduction

Patients with a history of cardiovascular disease (CVD) are considered to be at highest risk of recurrent major vascular events and mortality [1–3]. It has been estimated that over 300,000 Americans have recurrent coronary attacks annually [1]. Results from EUROASPIRE IV showed high rates of modifiable CV risk factors in patients with stable coronary heart disease as well as failure to achieve the recommended targets for all established risk factors in all patients; this might be due to a poor control of conventional risk factors [4, 5]. Additionally, less than one-half of the coronary patients have access to cardiac prevention and rehabilitation programs [5]. Recent statistics showed higher rates of secondary CVD events occurred in low-income countries [6, 7].

Although, in the overall population, the prevalence of conventional CVD risk factors has declined almost 50% during the past 3 decades [8], primary and recurrent CVD events are still a major health problem [9]. Additionally, this long period of decline in CHD mortality reversed in 2012, due to the offset by the substantial increases in the prevalence of obesity and diabetes [10]. Data also reveals that elimination of risk factors by healthy lifestyle coupled with multi drug regimens do much better than surgery procedures in prevention of CVD recurrence and determining the later prognosis of heart health [11]. Therefore, investigation of risk factors trends among CVD patients seems essential in order to prevent secondary CVD.

Several cross sectional studies showed high prevalence of CVD risk factors in patients with established CVD [12, 13]; however only a few studies have investigated the temporal trends of CVD risk factors among patients. To the best of our knowledge, no studies have been conducted among Middle East populations with high prevalence of CVD risk factors [14] and cardiovascular burdens [15].

Analyzing trends of targets achieved for CVD risk factors in our people with type 2 diabetes mellitus vs. non-diabetic population showed some improvement in controlling these factors among diabetic populations; however, almost over 60% of a diabetic population did not achieve the aim for LDL-C and blood pressure levels and over 70% of them still had central adiposity at end of study [16]. Recently we also investigated the impact of different risk factors for incidence of CVD/ all-cause mortality among a population free of CVD at baseline [9], and findings showed that hypercholesterolemia, low high density lipoprotein cholesterol (HDL-C), diabetes, hypertension, current smoking and central adiposity (for CVD events) and low educational level (for all-cause mortality) remained as significant risk factors and accounted for over 70% risk for CVD/ all-cause mortality events. [9]

In the current study, we planned to further our previous research by examining the secular trends of CVD risk factors among patients with prevalent CVD as a proxy for recurrent CVD during a decade long follow-up of a Middle Eastern cohort of the Tehran

## Methods

### Study subjects

Detailed descriptions of TLGS have been reported elsewhere [17]. Briefly, TLGS is a community-based prospective study conducted on a representative sample of district #13 of Tehran, the capital of Iran. Data collection was initiated in 1999–2001 (phase 1) and is ongoing on a triennial basis, designed to continue for at least 20 years. In the current study, data of subjects who participated in phase 2 (2002–2005) were used. All subjects completed a written consent after being informed about the general aspects of the work and the study was approved by the ethics committee of the Research Institute for Endocrine Sciences (RIES); the details of data collection of the study have been reported elsewhere [17].

**Fig 1:**
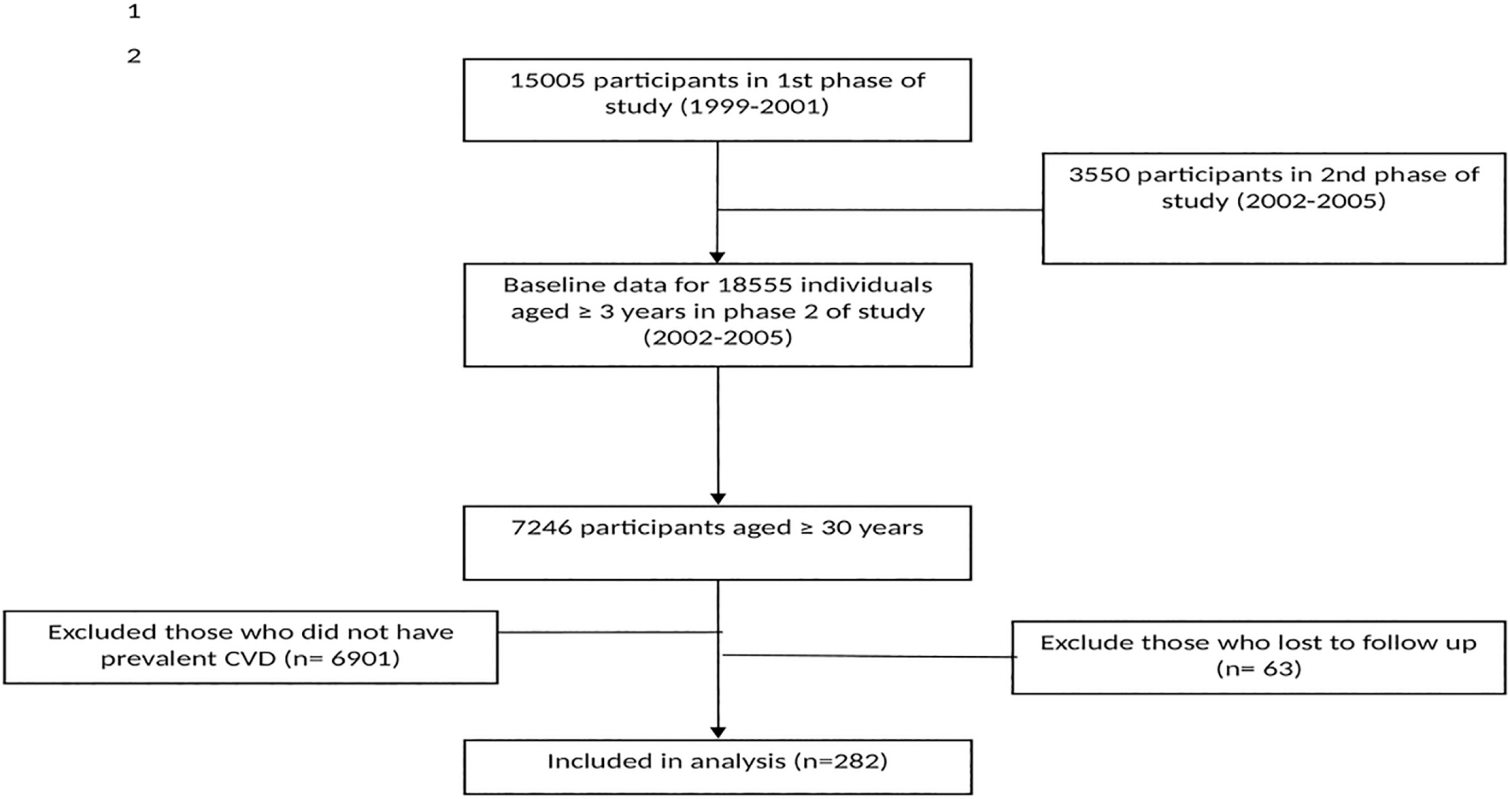
Study population.

Based on the revised and more detailed definition applied for CVD cases from phase 2, participants with known prevalent CVD, enrolled in phase 2 (n = 345) were selected to be followed in the current study. We excluded the participants who were lost to follow in any later phases (i.e., phase 3, 2005–2008, phase 4, 2008–2011 and phase 5, 2011–2014) after their enrollment (n = 63). Finally, 282 prevalent CVD patients (167 men and 115 women), mean age 60.76 years, were followed up for 12 years. (Fig 1)

### Clinical and anthropometric measurements

Subjects were interviewed by trained interviewers using pretested questionnaires. Information on age, sex, past medical history of CVD and smoking habits were collected. Anthropometric measures including weight, height and waist circumferences (WC) were recorded using standard protocols [18]. Body mass index (BMI) was calculated as weight in kilograms divided by height in square meters. Blood pressure was measured twice in a seated position after 15 minutes rest, using a standard mercury sphygmomanometer (calibrated by Iranian Institute of Standards and Industrial Researches) and the mean of the two blood pressures was considered as the participant’s blood pressure level.

### Laboratory measurements

After 12–14 hours of overnight fasting, a venous blood sample was drawn and centrifuged within 30–45 minutes of collection. All blood sampling was done between 7.00 and 9.00 A.M. and all measurements were completed on the day of sampling. Details of laboratory measurements including fasting plasma glucose (FPG), total cholesterol (TC), high density lipoprotein cholesterol (HDL-C) and triglycerides (TGs) are reported elsewhere [18, 19], non-high density lipoprotein cholesterol (Non-HDLC) was calculated by subtracting HDL-C from TC levels. Low density lipoprotein cholesterol (LDL-C) was calculated according to the modified Friedewald formula [20]. Estimated glomerular filtration rate (eGFR) was obtained using an equation derived from the modification of diet in the renal disease (MDRD) study. In this equation, eGFR is expressed as mL/min per 1.73 m2 and serum creatinine (Scr) as mg/dL [21]. Physical activity level was assessed with the Modifiable Activity Questionnaire (MAQ) from the 2nd phase. This questionnaire measures all three forms of activities including leisure time, job, and household activities in the past year [18]

### Definition of terms

Coronary heart disease (CHD) included cases of definite myocardial infarction (diagnostic electrocardiograph and biomarkers), probable myocardial infarction (positive electrocardiograph findings plus cardiac symptoms or signs plus missing biomarkers or positive electrocardiograph findings plus equivocal biomarkers) and angiographic proven CHD. CVD was defined as any CHD events plus fetal and non-fetal stroke.

Type 2 diabetes mellitus was ascertained among participants who had FPG ≥7 mmol/L or were on glucose lowering medications. General obesity was defined by BMI ≥30 kg/m^2^ and central adiposity was defined as WC ≥95 cm for both genders [22]. Among participants, systolic blood pressure (SBP) ≥140 mmHg or diastolic blood pressure (DBP) ≥90 mmHg [3] regardless of using anti-hypertensive medications were referred as hypertension (uncontrolled hypertension). Current smokers were defined as participants who were smoking cigarettes daily or occasionally as well as those who used water pipe or pipe.

High LDL-C was defined as ≥ 1.8102 mmol/L [23], high non-HDL-C as ≥ 2.586 mmol/L, low HDL-C as < 1.04 mmol/L for men and < 1.29 mmol/L for women and high TGs as ≥ 1.69 mmol/L [3].

Low physical activity (inactive) was defined as not achieving a minimum score of 600 MET (metabolic equivalent task)-minutes per week [24]. Chronic kidney disease (CKD) was defined as an estimated glomerular filtration rate <60 mL/min/1.73m2 [25].

### Statistical analyses

Baseline characteristics of participants have been reported as mean (standard deviation) for continuous variables and number (percentage) for the categorical variables. Only TGs have been reported as median (interquartile range).

Student's independent t-test was used for exploring differences in descriptive baseline characteristics (Mann-Whitney U test for TGs) between follow up and non-follow up populations.

The Generalized Estimation Equation (GEE) [26] method was used to assess secular longitudinal trends of continuous variables including BMI, WC, SBP, DBP, FPG, HDL-C, TG, TC, non-HDL-C, LDL-C and eGFR and for dichotomous variables, including general and central obesity, current smoking, CKD, low physical activity, and medication usage; separate GEE models with link function of logit and family of binomial were fitted for each variable.

To minimize selection bias, propensity scores (PS), the estimated probability of being lost to follow up based on individual characteristics at baseline was calculated. This measure was computed using maximum likelihood logistic regression analysis and was used for Inverse Probability Weighting [27]. Hence all, the entire baseline measures, including age, sex, FPG, TGs, TC, non-HDL-C, LDL-C, WC, SBP, DBP, BMI and eGFR were included in a logistic model as exposures with participation in the follow-up as the outcome; the probability of participation in follow-up was then estimated for every participants. The probability of participation in follow-up was used as a propensity score, which was added to the logistic models as a covariate; selection bias, therefore, probably did not affect our estimations. Since, the proportions of male to female participants differed from phase 2 to phase 5 during the follow up period, and also gender has some effect on the level of risk factors and may confound the results, in addition to age and PS, sex of each phase, as a covariate, was entered in models.

Interactions between sex and each variable were checked in a separate model, for which, we entered the cross-product term (interaction term) in a single model including both sex and each variable. Because of no significant interaction (all p-values≥ 0.104), results are reported among whole population to achieve statistical power. All analyses were done, using STATA statistical software (version 14 SE). P-value 0.05 is considered statistically significant.

## Results

The study population consisted of 282 prevalent CVD patients (167 men and 115 women) at baseline (i.e. phase 2, 2002–2005) with a mean (SD) age of 60.76 (± 9.49) years. Baseline characteristics of respondents and non-respondents participants were shown in Table 1, indicating no difference between risk factors in respondents and non-respondents except for FPG (6.48 in respondents vs. 7.49 mmol/L in non-respondents, p value = 0.01) and eGFR (65.84 in respondents vs. 61.83 (ml/min/1.73m^2^) in non-respondents, p value = 0.046).

**Table 1:** Baseline characteristics of respondents and non-respondents participants.

	Followed (n = 282)	Not followed (n = 63)	p-value
Age (years)	60.76(9.49)	62.31(11.31)	0.258
Sex (men)	167(40.8)	33(52.4)	0.320
BMI (kg/m^2^)	28.09(4.42)	27.74(4.93)	0.606
WC (cm)	97.40(9.93)	97.32(10.25)	0.958
SBP (mmHg)	130.98(21.11)	129.57(21.95)	0.637
DBP (mmHg)	78.35(11.75)	76.12(12.46)	0.179
FPG (mmol/L)	6.48(2.56)	7.49(3.54)	0.01
HDL-C (mmol/L)	0.97(0.24)	0.94(0.25)	0.434
TGs^a^ (mmol/L) median(IQR)	2.28(1.41)	2.47(1.98)	0.357
TC (mmol/L)	5.40(1.14)	5.46(1.08)	0.686
Non-HDL-C (mmol/L)	4.42(1.12)	4.51(1.10)	0.562
LDL-C (mmol/L)^b^	3.46(0.91)	3.49(0.83)	0.768
eGFR(ml/min/1.73m^2^)	65.84(13.53)	61.83(16.46)	0.046

^a^presented as median (interquartile range).

^b^values are presented as mean (SD) unless otherwise indicated

FPG, fasting plasma glucose; HDL-C, high-density lipoprotein cholesterol; TGs, triglycerides; TC, total cholesterol; Non-HDL-C, non-high-density lipoprotein cholesterol; LDL-C, low-density lipoprotein cholesterol; WC, waist circumference; SBP, systolic blood pressure; DBP, diastolic blood pressure; BMI, body mass index

**Fig 2:**
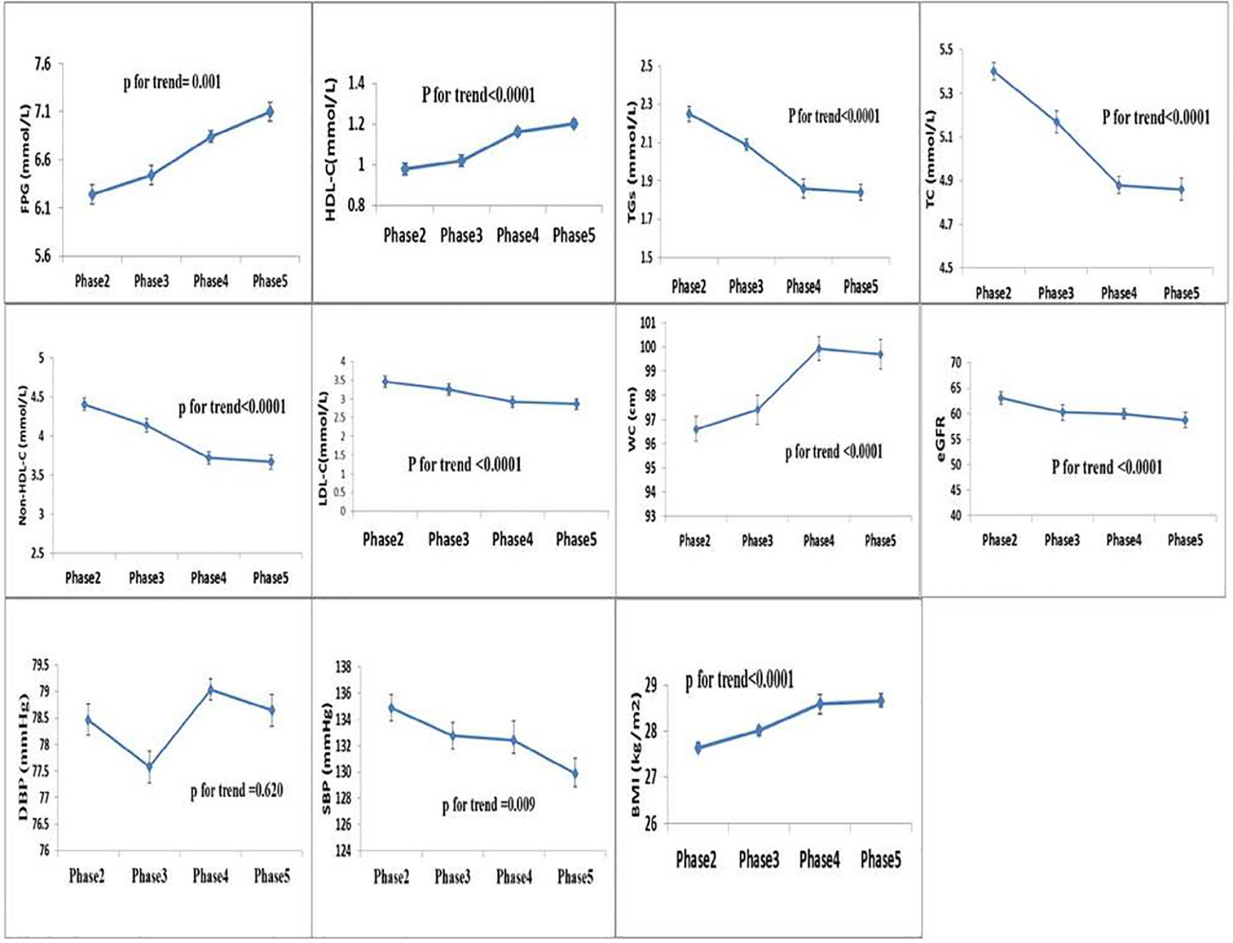
The trend of adjusted means of CVD risk factors in participants during the follow up. The follow up period of Tehran Lipid and Glucose Study included Phase 2 (2002–2005), Phase 3 (2005–2008), Phase 4 (2008–2011) and Phase 5 (2011–2014).

Table 2 illustrates the age, sex and PS adjusted means of CVD risk factors among the study population in each phase. Triglycerides, TC, non-HDL-C and LDL-C decreased significantly over the follow-up time from phase 2 to phase 5, (for TGs, 2.25 vs 1.84 mmol/l, for TC 5.40 vs 4.86 mmol/l and for LDL-C, 3.46 vs 2.86 mmol/l); however, HDL-C level increased from 0.98 to 1.20 mmol/l (all p values< 0.001). Additionally, levels of FPG, WC and BMI had significant increase from phase 2 to 5 (all p value < 0.001). On the other hand, SBP and eGFR levels decreased significantly during the follow up (for SBP, 134.88 mmHg in phase 2 vs. 129.86 mmHg in phase 5 and for eGFR, 63.11 ml/min/1.73m^2^ in phase 2 vs. 58.78 ml/min/1.73m^2^ in phase 5, all p values < 0.05). No more significant changes were observed in DBP. (Fig 2)

**Table 2:** Adjusted means of CVD risk factors in men and women participants in each phase; Tehran Lipid and Glucose Study.

	Phase 2 (2002–2005) (n = 282, men = 167)		Phase 3 (2005–2008) (n = 253, men = 148)		Phase 4 (2008–2011) (n = 225, men = 134)		Phase 5 (2011–2014) (n = 186, men = 109)		P-value for trend
	Mean	CI,95%	Mean	CI,95%	Mean	CI,95%	Mean	CI,95%	
BMI (kg/m^2^)	27.65	27.15–28.13	28.01	27.50–28.51	28.59	28.01–29.15	28.67	27.96–29.39	<0.0001
WC (cm)	96.61	95.41–97.80	97.41	96.21–98.61	99.93	98.57–101.28	99.69	97.96–101.43	<0.0001
SBP (mmHg)	134.88	132.40–137.36	132.73	130.12–135.34	132.38	129.58–135.17	129.86	126.58–133.14	0.009
DBP (mmHg)	78.47	77.08–79.86	77.58	76.11–79.06	79.04	77.46–80.61	78.65	76.90–80.40	0.620
FPG (mmol/L)	6.24	6.00–6.49	6.44	6.15–6.73	6.84	6.49–7.19	7.10	6.67–7.53	0.001
HDL-C (mmol/L)	0.98	0.95–1.01	1.02	0.99–1.05	1.16	1.12–1.19	1.20	1.16–1.25	<0.0001
TGs^a^ (mmol/L)median(IQR)	2.25	2.09–2.41	2.09	1.95–2.23	1.86	1.74–1.97	1.84	1.67–2.02	<0.0001
TC (mmol/L)	5.40	5.24–5.54	5.17	5.02–5.31	4.88	4.74–5.02	4.86	4.70–5.03	<0.0001
Non-HDL-C (mmol/L)	4.41	4.26–4.56	4.14	4.00–4.29	3.72	3.58–3.86	3.67	3.49–3.81	<0.0001
LDL-C (mmol/L)^b^	3.46	3.33–3.57	3.25	3.13–3.37	2.92	2.80–3.04	2.86	2.72–3.00	<0.0001
eGFR (ml/min/1.73m^2^)	63.11	61.76–64.46	60.25	58.76–61.73	59.93	58.29–61.57	58.78	56.88–60.69	<0.0001

The values adjusted for age, sex and propensity score.

^a^presented as median (interquartile range). FPG, fasting plasma glucose; HDL-C, high-density lipoprotein cholesterol; TGs, triglycerides; TC, total cholesterol; Non-HDL-C, non-high-density lipoprotein cholesterol; LDL-C, low-density lipoprotein cholesterol; WC, waist circumference; SBP, systolic blood pressure; DBP, diastolic blood pressure; BMI, body mass index; eGFR, estimated glomerular filtration rate

^b^formula for calculating LDL-C values as follows: LDL-C (mg/dl) = Non-HDL-C* 90%—TG *10%

values are presented as mean (SD) unless otherwise indicated

Table 3 illustrates the age, sex and PS adjusted prevalence of CVD risk factors in participants with prevalent CVD in each phase. The trends of dyslipidemia improved in significantly except for high LDL-C; prevalence for low HDL-C, hypertriglyceridemia, high non-HDL-C at baseline was 74.54%, 59.89%, 96.53% respectively and a trend which improved to reach 44.87%, 47.12%, and 96.06% respectively. Prevalence of general and central adiposity showed dramatic increase in our population (25.18% in phase 2 vs 41.32% in phase 5 for BMI≥30 kg/m2, 60.14% in phase 2 vs 66.74% in phase 5 for WC≥95 cm). Unfortunately a significant trend was observed for smoking status from 11.05% in phase 2 to 16.83% in phase 4 (p = 0.042). Significant increasing trends were observed in the consumption of anti-hypertensive, lipid and glucose lowering medications, but not for aspirin. Additionally, the prevalence of diabetes and low physical activity augmented significantly during our follow up from phases 2 to 5 (25.03 vs. 43.20%, and 43.74 vs. 50.32%, respectively). Furthermore, marginally significant trend was observed for the prevalence of CKD (44.16% in phase 2 vs. 51.65% in phase 5, p value = 0.054). However, no significant trends were seen for prevalence of hypertension (64.21 in phase 2 vs. 68.00 in phase 5, p value = 0.326).

**Table 3:** Adjusted prevalence of CVD risk factors in participants with prevalent CVD in each phase; Tehran Lipid and Glucose Study.

	Phase 2 (2002–2005) (n = 282, men = 167)	Phase 3 (2005–2008) (n = 253,men = 148)	Phase 4 (2008–2011) (n = 225,men = 134)	Phase 5 (2011–2014) (n = 186,men = 109)	P-value for trend
BMI≥30 kg/m^2^ (%)	25.18	31.67	36.36	41.32	<0.0001
WC≥95 cm (%)	60.14	62.55	70.35	66.74	0.038
Hypertension (%)	64.21	50.73	62.90	68.00	0.326
Low HDL-C (%)	74.54	71.30	47.73	44.87	<0.0001
High TGs (%)	59.89	56.12	46.33	47.12	0.001
High–non-HDL-C (%)	96.53	91.08	85.97	96.06	0.020
High LDL-C (%) ^a^	97.22	93.83	89.77	96.83	0.168
DM (%)	25.03	27.15	36.21	43.20	<0.0001
CKD (%)	44.16	52.28	50.91	51.65	0.054
Smoking (%)	11.05	12.27	12.67	16.83	0.042
Physically inactive (%)	43.74	36.13	50.12	50.32	0.037
Aspirin consumption (%)	60.53	56.40	62.37	60.08	0.845
Glucose-lowering medication use (%)	18.36	21.01	29.72	39.53	<0.0001
Lipid-lowering medication use (%)	21.12	26.11	35.25	47.44	<0.0001
Antihypertensive medication use (%)	41.31	17.56	45.80	54.09	<0.0001

The values adjusted for age, sex and propensity score. HDL-C, high-density lipoprotein cholesterol; TGs, triglycerides; Non-HDL-C, non-high-density lipoprotein cholesterol; LDL-C, low-density lipoprotein cholesterol; WC, waist circumference; BMI, body mass index

^a^ formula for calculating LDL-C values as follows: LDL-C (mg/dl) = Non-HDL-C* 90%—TG *10%

low HDL-C is defined as < 1.04 mmol/L for men and < 1.29 mmol/L for women, high triglycerides is defined as TG ≥ 1.69 mmol/L, high Non-HDL-C is defined as non-HDL-C ≥ 2.586 mmol/L or using lipid drug, high LDL-C as LDL-C ≥ 1.8102 mmol/L

CKD id defined as those with eGFR<60 mL/min/1.73 m2

Physically inactive is defined as achieving a minimum of 600 MET (metabolic equivalent task)-minutes per week

diabetes is defined as FPG ≥ 126 mg/dl (7 mmol/l) or taking any medication for diabetes

hypertension is defined as systolic blood pressure≥140 mmHg or diastolic blood pressure≥90 mmHg

## Discussion

During more than a decade long follow-up of a Middle Eastern population with prevalent CVD, an increasing trend was observed in the prevalence of general and central obesity, type 2 diabetes mellitus, CKD and physical inactivity. Despite a favorable trend for dyslipidemia, at the end of follow-up, about 97% of patients had levels of LDL-C ≥ 1.8102 mmol/L and over 50% did not report consumption of lipid lowering medication. Unfortunately, among CVD patients, an increasing trend from about 11% to 17% was highlighted for smoking whereas consumption of antiplatelet drugs among patients remained stable at a rate of about 60%.

It has been estimated that 92.1 million US adults have at least 1 type of CVD and almost 300,000 patients with a history of CVD are considered to have recurrent coronary attacks annually [1]. A study predominantly conducted in Iran reported that, of 4,735 patients who underwent coronary artery bypass surgery in Iran, 44.6% were referred and only 16.5% completed the cardiac rehabilitation program; completion rates were substantially lower in women than in men (15.6% versus 20.0%). This statistics showed that in line with our findings, CVD patients have poor control of CVD risk factors such as physical inactivity, diabetes, central obesity, dyslipidemia, smoking status, etc. [4].

Hypertension is one of the most important risk factors of primary and recurrent CVDs and its treatment is essential not only in relation to primary, but also for secondary prevention [28]. According to a scientific declaration of the American Heart Association, targeting blood pressure levels < 140/90 is recomnded for CVD patients with hypertension to prevent recurrent CVD [11]. Convincing evidence demonstrates that antihypertensive treatment and blood pressure reduction among patients with clinical history of CVD, with or without hypertension was associated with decreased risk of CVD events [29]. Although the mean level of SBP but not DBP decreased during our study, we did not observe a favorable trend for uncontrolled hypertension among Iranian CVD patients, findings in line with several analyses conducted in the United States [13, 30]. The transient decline observed in hypertension prevalence in 2005–2008, compared to 2002–2005 (50.73% vs. 64.21%, respectively) might be attributable to a cohort effect which causes patients to be more careful about their life style and nutritional habits (reducing salt intake) leading to better control of their hypertension, mostly at earlier phases of follow up. Despite the significant increase of consumption of anti-hypertensive medication, in our study at the last follow-up; only about 54% of patients used antihypertensive drugs, while the rest were not receiving any drugs. Furthermore, we should address the association between blood pressure and cardiovascular risks in secondary prevention, which is the influence of reverse causation, i.e.; a low attained blood pressure might be a consequence, not a cause [28].

Regarding obesity status, in our study significant increases in rates of general and central adiposity were observed, findings similar to those for general adiposity also reported among American patients with prevalent CVD [13]. Recently, we observed being overweight was associated with lower risk of recurrent CVD, whereas the presence of central adiposity was accompanied by a high risk [31]. Also systematic reviews of patients with coronary artery diseases or those undergoing percutaneous coronary intervention and cardiac surgery, have suggested an ‘obesity paradox’ whereby obesity appears to be protective [32–34]. However, this evidence should not be misinterpreted to recommend higher BMI as a negative risk factor for CVD patients, since reverse causality may be an important issue [35]. Therefore, as emphasized by American Heart Association and American College of Cardiology Foundation (AHA/ACCF), all patients should consistently be advised for weight maintenance or loss to reach BMI levels < 25 kg/m^2^ through regular physical activity and balanced caloric intake. Furthermore, lifestyle interventions should also be intensified to prevent abdominal obesity as well [3]. Unfortunately, our study revealed an unfavorable trend in the levels of physical inactivity, reaching over 50% in all patients.

Regarding incidence of CKD, among the general Iranian population, history of CVD was not found as an independent risk factor [36]. However, our results revealed a marginally significant increase from about 44% to 52% in the prevalence of CKD among CVD patients. Importantly, studies showed that the presence of CKD is an independent risk factor for primary as well as recurrent CVD events [37–40], and that the management of cardiovascular disease differs and is more complicated in patients with CKD [41].

In line with global diabetes trends [42], we showed an increasing trend in the prevalence of diabetes, i.e., the prevalence of diabetes increased from 25 to 43% among CVD patients. However, recently, among a general Iranian population of adults, we observed that history of CVD was not an independent risk factor for incidence of diabetes [43]; AHA/ACCF jointly recommend intensive risk factor modification for prevention of cardiovascular complications in patients with diabetes [3]. During a 7 year follow-up, we showed that the presence of both CHD and known diabetes were associated with an over 4–8 fold increased risk of CHD events among men and women, respectively [44].

The AHA/ACCF secondary prevention guidelines recommend low dose aspirin use for secondary CVD prevention, unless contraindicated with level (A) evidence [3]. Long-term aspirin use for secondary prevention of atherothrombotic events reduces the yearly risk of serious vascular events by about a quarter [45, 46]. Recently, it was reported that among an American population with prevalent CVD, aspirin usage for secondary CVD prevention was declined to an average annual change rate of -3.6% [47]. At the end of follow up, in our study population, about 40% of CVD cases did not report aspirin usage. Therefore, health policy makers and physician should advocate the routine use of aspirin in primary CVD patients, assuring them about its confirmed benefits in preventing secondary diseases.

Regarding the importance of cholesterol lowering for secondary CVD prevention [48], all patients with clinical atherosclerotic diseases require high dose statin therapy, regardless of baseline LDL-C level or the presence of other CVD risk factors, to reduce LDL cholesterol levels by ≥50%. Also evidence from trials has suggested that lowering LDL-C to 1.8 mmol/L (<70 mg/dL) is associated with a lower risk of recurrent CVD. In the current study, similar to our pervious finding, we observed a favorable trend for dyslipidemia, which seems is due to the fact that of usage of lipid lowering medication increased > two folds among patients during follow-up. On the other hand, we previously showed that over 30% of Iranian families are now consuming less hydrogenated oil than they did in the past [49, 50]. However, still more than 50% of cases did not report any usage of lipid lowering medications. Furthermore, the prevalence of high LDL-C at year 2014 is still more than 96%; which might be attributable to poor compliance of medication consumption [8] as well as inadequate dosage for statin medications [51, 52].

The most recent guidelines recommend that tobacco users quit smoking completely for prevention of recurrent CVD [3]. However, among Tehranian adult CVD patients, the prevalence of current smoking has increased from 11 to > 16% during the follow up in our study. Recently, we showed that current smoking among CVD cases was associated with 95% higher risk for recurrent events [31], finding in line with Mayo clinic PCI Registry study, which showed that despite improvement in the management of hypertension and lipid profiles, no favorable trend was observed following cessation of smoking among patients [30]. However, among American patients with history of myocardial diseases and stroke during 1988–1994 and 1999–2002, the level of current smoking decreased from 30.6 to 23.3%, which was marginally significant [13]. Since quitting smoking among patients with prevalent CHD was associated with about 36% risk reduction for mortality events, increasing education levels/knowledge and awareness in communities and implementing interventions seems essential to ban smoking among these patients [53]. Gigliotti et al. analyzed smokers’ reactions to hypothetical cigarettes price increases in Brazil and found out that 40–59 and 60 or more age groups had relatively the lowest tendency to quit or lower their smoking frequency [54]. Considering the fact that most primary CVD patients in our study were over 50 years old, and this group have the highest probability of becoming long term smokers, since most of them are financially more stable than younger populations, cigarette taxation might overall be a key policy. However, the effect of banning cigarette smoking among CVD patients is controversial and requires further research.

The current study highlights the need for urgent implementation of multicomponent interventions to control CVD risk factors among these patients. Multicomponent Interventions could include of using personal or family counselling and education, with or without pharmacological treatments to control hypertension, diabetes and hyperlipidemia and also the availability of healthier foods (with low fat and low salt) decrease obesity in primary CVD patients [55]. Due to the fact that urban air pollution is a major health risk in several large Iranian cities [56], the government can play a more effective role by ensuring easy access to appropriate exercise space (like sports complexes etc.) for various physical activities for CVD patients in every district. Maybe the free use of leisure facilities and sports complexes for CVD patients or elderly populations could encourage them to exercise more frequently [57]. Furthermore, some workplace physical activity interventions would also improve health and modify multiple risk factors such as disturbed lipid levels and central adiposity [58].

As strengths, we used the data of an ongoing prospective population based cohort study rather than clinic or hospital based data to assess the trends of CVD risk factors overtime. A relatively long period of follow up in addition to using standardized measurement methods by trained health professionals rather than self-reported measures increases both the accuracy and reliability of our reports. Our findings, however, need to be interpreted in the light of the study’s limitations. First, our study actually shows an optimistic picture for trend of CVD risk factors among CVD patients since inclusion of subjects in an ongoing study can increase the level of attention paid to controlling their health risks i.e. cohort effect. Therefore, the burden of measured risk factors is much higher in the context of the community. Second, our study is subject to the survival bias as the subjects with very high levels of risk factors could be lost in successive phases due to death from CVD events or other complications. Therefore, we entered PS as a covariate to our data analysis to reduce selection bias; however, some residual confounding due to aging of the study cohort does exist. Third, regarding our sample size, it was not possible to sex-stratify our data analysis, although no interaction was found between CVD risk factors and sex. Forth, the PA questionnaire is still self-reported and may subject to bias. However, in line with our findings, a national study conducted among Iranian adults ≥ 25 years by using global Physical activity questionnaire (GPAQ), which endorsed by WHO, showed Iranian populations, particularly females, became less active during the survey period (2007–2011) [59]. And the last, but not least, the population studied was of Persian ancestry, because of which, our results might not be directly extrapolated to other populations.

To conclude, during a long term follow up, except for lipid profile status, dangerous trends for the CVD risk factors among Iranian CVD patients were demonstrated, which be a harbinger for high rates of CVD mortality. These findings highlight the need for urgent implementation of multicomponent interventions to control CVD risk factors among these patients.

## Supporting information

S1 File Supporting data. (DTA)
